# A Deciduous Man

**Published:** 1891-03

**Authors:** 


### A Deciduous Man.

At a recent meeting of the Chicago Medical Society Dr. J. Frank reported a case where a man every July shed his skin. He was taken with feverish tremors, increasing almost to paroxysms. He undressed, lay down, and within a few minutes the skin of the chest began to turn red. The redness rapidly extended over the entire skin, and the feverish tremors continued uninterruptedly for about twelve hours. Then he arose, dressed, and walked about in perfect health. The skin now commenced to peal, and ten hours later it began to come off in great patches. From the arms and legs it could be pulled off exactly like gloves or stockings. As the old skin came away a new epidermis, as soft and pink as a baby's was revealed. This new skin was very sensitive; the patient had to wear softened gloves and moccasins for about a week. After the old cuticle had been entirely removed, the finger and toe nails began to drop off—new nails literally crowding them out. Finally the change was complete—the man had a new skin and a new outfit of nails and was ready to return to the mines. The shedding began in his first year and recurred every July thereafter.—*Medical Record.*

"So Much a Foot."—A bran-new graduate, fresh from the parting embraces of his alma mater, was called to attend an old lady suffering from tape worm. Having relieved her of the parasite he sent in an account of 10s 6d which the patient thought exorbitant, and asked for particulars.

These were given in the following terms: "For delivering you of a tapeworm 10½ feet long at a shilling a foot, 10s 6d."—*Medical Press.*

A Bad Field for Nostrums.—There is said to be a law in Bulgaria to the effect that if a patent medicine which is advertised to cure a certain malady fails to do so, the vender of the remedy is liable for damages, and may also be sent to prison for a limited period of time as a punishment for publishing an untruth to the injury of the public.

